# Vitamin D and juvenile idiopathic arthritis

**DOI:** 10.1186/s12969-018-0250-0

**Published:** 2018-05-16

**Authors:** Sarah L. Finch, Alan M. Rosenberg, Hassan Vatanparast

**Affiliations:** 1 College of Pharmacy and Nutrition, University of Saskatchewan, Saskatoon, Canada; 2 Department of Pediatrics, University of Saskatchewan, Saskatoon, Canada; 3 College of Pharmacy & Nutrition and School of Public Health, University of Saskatchewan, 104 Clinic Place, Saskatoon, SK S7N 2Z4 Canada

**Keywords:** Vitamin D, Childhood arthritis, Juvenile idiopathic arthritis

## Abstract

**Background:**

Vitamin D has been implicated in the pathogenesis of autoimmune diseases. While the roles of vitamin D in other autoimmune diseases have been investigated, less is known about the role of vitamin D in chronic childhood arthritis.

**Main body:**

This review summarizes and evaluates evidence relating to 25-hydroxyvitamin D (25(OH)D) and chronic childhood arthritis. A scoping literature review was conducted using Ovid Medline, Ovid Embase, Cumulative Index to Nursing and Allied Health Literature, Web of Science and Scopus. Further, we geo-mapped the results of the studies to identify the patterns of the association between vitamin D and chronic childhood arthritis across the globe. Of 38 studies reporting 25(OH)D concentrations in childhood chronic arthritis, 32 (84.2%) reported that a significant number of children had suboptimal (< 75 nmol/L) status.

**Conclusion:**

The data indicate suboptimal vitamin D status in children with chronic arthritis. Further, the association between low vitamin D and increased arthritis activity follow a north-south geographical gradient.

## Background

Arthritis is among the most common chronic diseases in children. Juvenile Idiopathic Arthritis (JIA) is the current nomenclature applied to denote a group of clinically distinguishable subsets that share chronic, childhood-onset arthritis of unknown cause as a unifying feature. The etiologies of JIA are unknown and the pathogeneses unclear but are likely multifactorial. Among epidemiologic studies there is substantial variability in the frequencies with which JIA and its respective subtypes are reported to occur; chronic arthritis prevalence rates range from 0.07 to 4.01/1000 children and annual incidences from 0.008 to 0.226/1000 children [1]. Putative explanations for the disparities in reported juvenile arthritis prevalence rates include, as examples, differences in diagnostic criteria applied (specifically, JIA or the earlier Juvenile Rheumatoid Arthritis (JRA) [2] or Juvenile Chronic Arthritis (JCA) [3] classification systems) and in case ascertainment methods.

While methodologic inconsistencies among JIA epidemiologic studies might account for perceived prevalence differences, actual differences might occur as a consequence of genetic, ethnic, environmental, and lifestyle influences. Vitamin D status is potentially governed by these same factors; vitamin D receptor genotype, ethnically related skin tone and clothing, environmental variations in exposure to ultraviolet B radiation relating to the latitude of residence and season, and vitamin D nutritional intake are factors that modulate vitamin D concentrations.

As an immune and inflammatory mediator, vitamin D is implicated in the pathogenesis of autoimmune diseases including, as examples, multiple sclerosis, type 1 diabetes, rheumatoid arthritis, Crohn’s disease, and chronic childhood arthritis [4–6]. Cells involved in innate and adaptive immune responses such as macrophages, dendritic cells, T cells, and B cells express enzymes required to activate and respond to vitamin D [7–9]. Cytochrome p450 27B1 (CYP27B1) is the enzyme required to synthesize 1,25-dihydroxyvitamin D (1,25(OH)2D), the active form of vitamin D, from circulating 25-hydroxyvitamin D (25(OH)D). The actions of 1,25(OH)2D are mediated by its binding to the vitamin D receptor (VDR), a nuclear transcription factor. VDR then binds to the Vitamin D Response Element (VDRE), a genetic sequence located in the promotor region of genes regulated by vitamin D [7–9]. Vitamin D tends to suppress the immune response [6]. Consequently, low vitamin D concentrations are associated with an increase in pro-inflammatory mediators and more active disease [6, 10] consistent, for example, with the observation that low serum 25(OH)D is associated with increased disease activity in rheumatoid arthritis [11].

Reports of relationships between vitamin D and chronic childhood arthritis are derived from studies having different methodologic approaches, originating from multiple geographic regions, and comprising demographically disparate populations. Since the last analysis of these reports in 2013 [12] the number of studies reporting 25(OH)D concentrations in children with chronic arthritis have increased from 14 to 38. An updated, systematic analysis of pertinent literature should help to further refine understanding of the relationships between vitamin D and juvenile arthritis, contribute to optimizing management of vitamin D status in children with arthritis, and clarify vitamin D’s potential role in mediating disease pathogenesis.

Scoping reviews are methodologic approaches for thoroughly distilling and synthesizing information derived from different studies having varied designs. The purposes of scoping reviews are to not only capture key concepts that can guide care but also to recognize knowledge gaps that can inspire future research priorities [13].

Although nomenclature applied to chronic childhood arthritis classification systems has changed over the years, JIA is the current terminology. For clarity, this review will hereafter use the term JIA to encompass the forms of chronic childhood arthritis also included in the JCA and JRA classification systems. However, in this review, when quoting the literature, we use the terminology for chronic childhood arthritis (JCA, JRA, or JIA) that was applicable at the time the cited reference was published [14].

Here we report the results of a vitamin D- JIA scoping review that summarizes, synthesizes, evaluates, and interprets pertinent evidence from the literature to address the following research questions: 1) What is the relationship between vitamin D status and the occurrence of JIA? 2) What is the relationship between vitamin D status and childhood arthritis activity? 3) What is the relationship between vitamin D status in JIA and medication use? 4) What is the relationship between vitamin D status and geographic and demographic characteristics in children with JIA?

## Methodology

To ensure a comprehensive literature scan, this scoping review applied the iterative methodological framework escribed by Arksey and O’Malley, with refinements by Levac et al. and Colquhoun et al. [14–16]. Five biomedical literature search engines were accessed, in the following sequence: Medline (using Ovid), Embase (using Ovid), Cumulative Index to Nursing and Allied Health Literature (CINHAL), Web of Science, and Scopus. Reference lists within each of the publications retrieved from the web-based searches were scanned to ensure that no relevant citations were missed. Medical Subject Heading (MeSH) terms used for retrieval were “vitamin D” and “juvenile arthritis”. The search term “juvenile arthritis” was general enough to capture citations that referred to JCA, JRA and JIA classification terms (Table 1). Search results, which included published articles, letters, and abstracts from conference proceedings, were collated and duplicates of articles removed. Publication titles and abstracts were then screened for relevance to the subject of vitamin D in children with idiopathic chronic arthritis as defined by JIA, JCA, or JRA classification criteria [2, 3, 17]. The full texts of relevant articles were then reviewed.

**Table 1:** Comparison of classification systems of chronic childhood arthritis^a^

	American College of Rheumatology 1977 [2]	European League Against Rheumatism 1978 [3]	International League Against Rheumatism 1994 and 2001 [17]
Classification Title	Juvenile Rheumatoid Arthritis	Juvenile Chronic Arthritis	Juvenile Idiopathic Arthritis
Symptom duration	Minimum 6 weeks	Minimum 3 month	Minimum 6 weeks
Subtypes	Systemic	Systemic	Systemic
	Polyarticular	Polyarticular	Polyarthritis RF negative
		JRA (RF positive Polyarticular)	Polyarthritis RF positive
	Pauciarticular	Pauciarticular	Oligoarthritis
			Persistent
			Extended
		Juvenile psoriatic	Psoriatic arthritis
		Juvenile ankylosing spondylitis	Enthesitis-related arthritis
		Arthritis associated with inflammatory bowel disease	
			Undifferentiated arthritis

^a^Prior to 1997, two chronic childhood arthritis classification systems were used. The American College of Rheumatology (ACR) [2] classification criteria referred to chronic childhood arthritis as Juvenile Rheumatoid Arthritis (JRA) and the European League Against Rheumatism (EULAR) applied the term Juvenile Chronic Arthritis (JCA) [3]. Differences between the two classification systems hindered exchange and comparison of data between the two systems [61]. To reconcile differences between ACR and EULAR criteria, the International League Against Rheumatism (ILAR) JIA criteria were introduced. This table provides a comparison of diagnostic criteria [17]. The ILAR classification system defines JIA as all forms of inflammation of one or more joints beginning in children younger than age16 years [17]. JIA is further classified into seven categories based on inclusion and exclusion criteria according to features present within the first six months of disease**.** The seventh category includes those who do not fit into one category, meet criteria for more than one category, or have exclusion criteria that preclude assigning a category

**Fig. 1:**
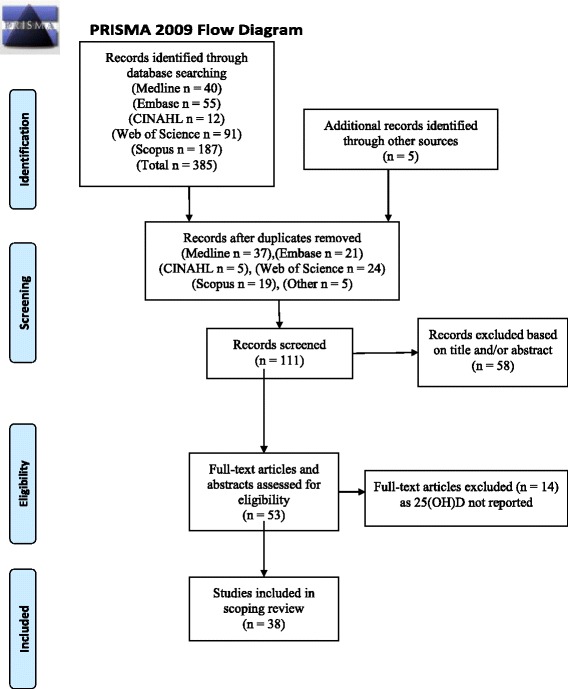
Articles identified from the retrieved publications reference lists are identified as “other” in the flow diagram

Inclusion criteria for the review were 1) study conducted in humans, 2) 25(OH)D concentrations reported, 3) participants having a diagnosis of JCA, JRA, or JIA, and 4) JCA, JRA, JIA diagnosis without an associated coexistent autoimmune disease. Exclusion criteria were 1) study conducted in animals, 2) the presence of an associated autoimmune disease 3) pregnant or lactating subjects, 4) 25(OH)D concentrations not reported), and 5) review articles. The process applied to identify eligible articles for the review is shown in Fig. 1 [18]. Articles in any language were eligible; however, no non-English language articles without at least an English abstract were found. From all relevant articles retrieved the following information was extracted: juvenile arthritis classification system (JCA, JRA, or JIA); sample size; sex ratio; patient age (at baseline or time of study if the study was cross-sectional); geographic location (country, city, and latitude and longitude), year of study; 25(OH)D concentration; study conclusion; and, if applicable, control group sample size, characteristics, and 25(OH)D concentration. The latitudes and longitudes reported were that of the city where the study was conducted; if unavailable, the province, state or region’s center latitude was used and, as a last resort, the country’s central latitude was used. 25(OH)D status by season of measurement was not reported in any of the articles found and therefore could not be considered.

Geographic Information Systems (GIS) mapping was performed using ArcGIS version 10.4 to visualize studies by juvenile arthritis classification, 25(OH)D status, location, and latitude. For all reported studies, an additional map comparing the difference in 25(OH)D concentration between those reporting active versus inactive disease was made.

### Defining vitamin D status

Vitamin D deficiency is defined as a serum 25(OH)D concentration less than 30 nmol/L, a 25(OH)D concentration between 30 and 50 nmol/L is considered insufficient, greater than 50 nmol/L is considered sufficient and a 25(OH)D status greater than 125 nmol/L is considered at risk of adverse effects [19]. These values are based on the Institute of Medicine (IOM) review of published research focused on determining the optimal vitamin D concentration for maximal calcium absorption, prevention of rickets, reduction of fracture risk and prevention of osteomalacia in healthy populations [19]. In the most recent review of vitamin D requirements, the IOM concluded that there was inadequate information to make intake recommendations in relation to other biologic roles of vitamin D [19]. The current recommended dietary allowances (RAD) of vitamin D are 400 IU from 0 to 12 months of age and 600 IU per day from 1 to 18 years of age [19].

The Endocrine Society has published clinical practice guidelines for patients at risk of vitamin D deficiency [20]. The Society recommends that at-risk populations, including “obese children and adults and children and adults on anticonvulsant medications, glucocorticoids, antifungals such as ketoconazole, and medications for acquired immune deficiency syndrome be given at least two to three times more vitamin D for their age group to satisfy their body’s vitamin D requirement” [20]. The optimal 25(OH)D concentration suggested by the Endocrine Society is 75 nmol/L. To meet this concentration it is recommended that 400–1000 IU be given between 0 to 12 months of age, 600–1000 IU per day from 1 to 8 years of age, and 1500–2000 IU for children between ages 9–18 years [20]. These recommendations, however, are not specific for children with chronic arthritis.

## Results of scoping review and discussion

Considerations when evaluating the role of vitamin D in JIA include vitamin D requirements for this population and the role that vitamin D plays in disease activity. Using the specified MeSH search terms (vitamin D and childhood arthritis), 386 reports (full-text articles, conference abstracts, and letters to the editor) were identified. Thirty-eight studies met the inclusion criteria and are the subject of this review (Table 2). One meta-analysis reported cumulative 25(OH)D concentrations from fourteen studies comprising children with JIA, JCA, and JRA and other rheumatic conditions; this meta-analysis was not included in our scoping review but is referenced in the discussion [12]. This present review summarizes accumulated evidence on vitamin D and chronic childhood arthritis by disease activity and latitude. Additionally, this study provides new information about differences in 25(OH)D status between healthy controls and children with JIA.

**Table 2:** Summary of current literature of 25(OH)D status and chronic childhood arthritis

Study Location and Reference	Disease	Sample size (number female)	Age (years) Mean ± SD or range	25(OH)D (nmol/L) Mean ± SD or range	Results relating to vitamin D	Control Group Results	Vitamin D Intake
Study Design: Meta-Analysis							
Meta-Analysis Nisar et al. 2013 [12]	JRA JCA & JIA	*n* = 529	0–18	61.4	Mean of 14 studies 61.4 nmol/L (Range 28.7–139.8) prevalence reported from 3 studies 82% insufficient.		
Study Design: Randomized Controlled Trial							
Cincinnati, Ohio USA Stark et al. 2006 40°N [45]	JRA	*n* = 49	4–10 y	79.9 ± 25.0 (39.9–142.3)	Behaviour intervention to increase calcium intake successful.		
Ioannina, Greece Siampoulou et al. 2001 39°N [40]	JIA	n = 10 (6F)	13.1 ± 2.5	53.9 ± 8.5	All patients were vitamin D replete 25(OH)D > 17.5 nmol/L, most were measured between February to May.		
Missouri, USA Hillman et al. 2008 37°N [54]	Children with arthritis	*n* = 18	3–15	82.1 ± 38.7	Supplemental vitamin D improved status, but supplemental vitamin D or calcium did not improve bone mass.		
Kansas, USA Warady et al. 1994 39°N [55]	Rheumatic disease (6 with JRA)	*n* = 10 (7F) 6 JRA	13.(10.9–18.0)	70.1 ± 21.2	Children with rheumatic disease would benefit from receiving calcium and vitamin D supplements.		
Illinois, USA Reed et al. 1991 40°N [46]	JRA	*n* = 13 (12F)	5–18	70.0 ± 40	Vitamin D may help prevent bone loss in children with active disease.		
Study Design: Case-Control							
Istanbul, Turkey Dagdeviren- Cakir et al. 2016 41°N [21]	JIA	Active disease: *n* = 64 (41) Remission: 53(35)	Active disease: 9.7 ± 4.3 Remission: 9.8 ± 4.3	Active disease: 46.5 ± 23.0 Remission: 47.3 ± 27.5	Vitamin D concentrations in children with JIA were significantly lower than healthy children. Of those who were measured while in remission there was no difference in 25(OH)D concentrations.	Healthy control *n* = 100 66.8 ± 26.6 nmol/L.	
Mexico city, Mexico Hernandez Rosiles et al. 2015 19°N [39]	JIA	*n* = 37 (27)	12.5 ± 3.1	55.0 ± 13.9	No difference between children with JIA and controls.	Healthy controls *n* = 79 59.0 ± 7.7 nmol/L.	
Fortaleza, Brazil De Sousa- Studart et al. 2015 3°S [22]	JIA	*n* = 51 (31 F)	13.4 ± 4	55.4 ± 25.0	25(OH)D similar for disease activity status, JIA category, and arthritis severity measure.	Age sex-matched controls 25(OH)D, 75.9 ± 14.0 nmol/L.	
Hangzhou, China (article in Chinese, English Abstract) Wang et al. 2015 30°N [60]	JIA	*n* = 53	Not reported	Median 42.6	A significant difference between JIA and control 25(OH)D *p* < 0.01. No correlation between 25(OH)D and JIA subtypes, ACR pediatric 30, CRP or ESR.	Control *n* = 106 25(OH)D 49.9 nmol/L.	
Florence, Italy Stagi et al. (2014) 43°N [23]	JIA	n = 152 (115F)	16. ± 7.4	54.4 ± 20.5	JIA had reduced 25(OH)D and higher PTH compared to controls. Active disease or frequent flare-ups resulted in lower vitamin D than non-active and no frequent flare-ups.	Control group 25(OH)D 74.4 ± 28.0 nmol/L *p* < 0.005.	Intake JIA 164 ± 84 IU/day control 160 ± 72 IU/day.
New Delhi, India Dey et al. 2014 28°N [31]	JIA	n = 35	3–16	22.0 ± 18.0	Decreased dietary intake of vitamin D and calcium, decreased weight bearing physical activity and sunlight exposure were the major factors for low BMD. Duration of disease 2.30 ± 1.91 yrs.	Age sex-matched controls 25(OH)D 37.9 ± 10.0 nmol/L significant difference.	Intake JIA 123 ± 53.6 (50–207) control 309 ± 62.38 (213–387) IU/day.
Lodz, Poland Szyamanska-Kaluza et al. 2013 51°N [24]	JIA	*n* = 50 (40)	9.4 ± 5.52	43.4 ± 21.1	Vitamin D deficiency is common in this population. No correlation between disease activity, type of JIA or metabolites of vitamin D.	Control *n* = 28 Age, gender matched, hospitalized children 43.4 ± 40.7 nmol/L.	
Sao Paulo, Brazil Munekata et al. 2013 23°S [25]	JIA- polyarticular	*n* = 30 (23F)	14 (4–20)	64.1 ± 21.6	High frequency of 25(OH)D deficiency in both control and JIA groups; no difference between the two. No association of 25(OH)D with disease activity.	Control group age-sex matched (n = 30). 16 non-Caucasian; mean disease duration 5y (1–12) control 2 5(OH)D 67.2 ± 19.0 nmol/L.	
Oslo, Norway Lien et al. 2005 59°N [26]	JIA	*n* = 108	6 to 18	49.7 ± 16.5	No difference in 25(OH)D between control and JIA groups.	Control n = 108 25(OH)D 50.4 ± 8.1 nmol/L D	Intake JIA 164 ± 84 IU control 160 ± 72 IU
London, UK Rooney et al. 2000 51°N [49]	JCA	*n* = 34 (23F)	9.2 (4.6–13.6)	Estimated from graph 45 (14.5–62.5)	Vitamin D status was significantly lower in JCA patients than age-matched controls before treatment, Steroid-treated children have low vitamin D. All but three children received corticosteroids.	Control group 25(OH)D estimate 75 nmol/L.	
Florence, Italy Falcini et al. 1998 43°N [48]	JCA	n = 47 (34)	15 months-12 years(7.13 ± 4.1)	61.4 ± 20.5	The lower serum concentrations of osteocalcin in active disease support the hypothesis that both bone formation and resorption are reduced in JRA	Controls *n* = 47 25(OH)D 56.7 ± 21.5 nmol/L.	
Missouri, USA Pepmueller et al. 1996 37°N [43]	JRA	*n* = 41	4–18.5	45.7 ± 23.5	Suggest an association between decreased bone mineralization in JRA and low bone formation that is related to disease severity.	Control *n* = 62 65.5 ± 23.5 nmol/L significant difference.	Vitamin D intake in JRA 464 ± 262 IU Intake of controls not reported.
Ioannina, Greece Tzoufi et al. 1994 39°N [50]	JCA	n = 35 (14)	8.8 ± 4.1	39.8 ± 20.5	Disease activity of JCA appears to be associated with lower vitamin D.	Mean disease duration 3.4 years Control *n* = 15 25(OH)D 68.1 ± 15.5 nmol/L control group taking corticosteroids n = 4 25(OH)D 51.4 ± 24.5 nmol/L.	
Missouri, USA Hillman et al. (1994) 37°N [47]	JRA	*n* = 44 (28)	11.8 ± 3.8	66.6 ± 26.7	Lower bone mineral content and bone biomarkers in JRA patients that controls but higher vitamin D in JRA.	N = 37 controls 25(OH)D 53.2 ± 18.7 nmol/L.	
Milano, Italy Bianchi et al. 1990 45°N [41]	JIA	*n* = 36 (64%)	9.96 (5–17)	45.9 nmol/L Reported from Nissar et al. Review	Suggests severe JRA has an influence on bone mass possibly mediated by a decrease in active vitamin D metabolites.	Study duration one year, controls only measured at baseline 25(OH)D 92 ± 17.5 nmol/L *N* = 20	
Huddinge, Sweden Johansson et al. 1986 59°N [52]	JCA	26 (all female)	11–16	63.2 ± 36.4	Statistically lower than controls, however no evidence of deficiency.	Healthy controls n = 28 76.2 ± 28.0 nmol/L	
Study Design: Cross Sectional							
Chongqing, China Tang & Mingyue Conference Abstract 2016 29°N [27]	JIA	*n* = 76 (36)	8.49 ± 3.09	52.8 ± 15.3 nmol/L	JIA patients have reduced serum 25(OH)D3, particularly those with active disease or/and using glucocorticoid.		
Riyahad, Saudi Arabia Alhomaidah et al. 2016 24°N [28]	JIA	*n* = 22 (13)	12.4	14 > 75 nmol/L 8 < 75 nmol/L	Vitamin D insufficiency is frequent in children with JIA.		
Bialystok, Poland (abstract only) Goralczyk et al. 2015 53°N [29]	JIA	*n* = 189 (113)	3–17.7	40.6 ± 23.5	67% 25(OH)D < 50 nmol/L. Obese children had significantly reduced 25(OH)D compared to normal weight peers. Negative relationship between MTX use and 25(OH)D.		
Oporto, Portugal Peixoto et al. 2013 Conference Abstract 41°N [30]	JIA	*n* = 40 (31)	22.3 (4–63)	10 > 75 nmol/L, 19 between 50 and 75 nmol/L, 11 < 20 nmol/L	Prevalence of vitamin D deficiency/insufficiency among JIA patients is very high.		
Antalya, Turkey Comak et al. 2014 36°N [32]	JIA	n = 47 (29)	9.3 ± 3.9	44.2 ± 29.0	Only 27.7% patients had 25(OH)D > 50 nmol/L. There was a significant negative correlation between vitamin D concentration and disease activity (*p* = 0.01, *r* = − 0,37).		
Salé, Morocco Bouaddi et al. (2014) 34°N [33]	JIA	n = 40 (18)	11 ± 4.23	55.4 ± 27.2	25(OH)D < 75 nmol/L in 75% of sample. Poly arthritis and oligoarthritis 25(OH)D status negatively associated with disease activity in univariate but not multivariate analysis.	Median disease duration two years.	
Helinski, Finland Miettinen et al. 2013 Letter to the Editor 60°N [34]	JIA	*n* = 136	1–18	M: 63.9 ± 18.0 F: 62.9 ± 20.0	Suggest that JIA subtype may be associated with 25(OH)D concentration in female patients. Seasonal difference with female patients.		
CambridgeUK Nisar et al. 2013 Conference Abstract 52°N [35]	JIA	n = 37 (31)	0–10 (n = 13), 11–20 (*n* = 12) and > 21 years (n = 12)	49.6 nmol/L (range 13.2–112.0 nmol/L).	Half of patients with JIA have low Vitamin D levels which are inversely related to disease activity and disease duration.		
Boston, USA Pelajo et al. 2012 42°N [36]	JIA	*n* = 154 (61%)	10.6	72.9 ± 23.0	13% deficient, 42% insufficient. Age, ethnicity, season, BMI associated with 25(OH)D but not vitamin D deficiency.No association with whole sample; small negative association for new onset JIA; mean time since onset 28 months.		
Helinski, Finland Markula-Patjas et al. 2012 60°N [37]	JIA	n = 50 (41)	14.8 (7.0–18.7)	53 nmol/L (20–95 nmol/L)	62% sufficient, 24% insufficient and 14% deficient.		52% taking vitamin D supplement % of DRI median and IQR 187 (57,331).
Saskatchewan, Canada McNally et al. 2009 52°N [59]	Pediatric arthralgia	*n* = 730 25(OH)D *n* = 73	< 18	59.9	Significantly more reported fall and winter as season of onset – more referrals from northern SK 40% < 50 nmol/L 42% 50–75 nmol/L Association between psychological stress, school absenteeism vitamin D insufficiency and arthralgia.		
Helinski, Finland Valta et al. 2007 60°N	JIA Glucocorticoid treated	n = 62 (43)	Median 11.8 (4.6–17.9	Median 49 nmol/L 16 (23%) ≤ 37.5 nmol/L	Osteoporosis is a concern in glucocorticoid treated children with JIA.		32% prescribed 400–800 IU vitamin D daily Mean intake 316 IU (range 44–1204 IU).
Ohio, USA Henderson et al. 1997 40°N [44]	JRA	*n* = 48 (37)	8.1 ± 1.9	89.4 ± 28.7	Serum 1,25-dihydroxyvitamin D concentrations were able to accurately segregate 79.6% of the JRA subjects into either the low or normal BMD groups.		%RDA 87.6 ± 52.7.
Illinois, USA Reed et al. 1993 40°N [42]	JRA	*n* = 27 (23)	2.9–16	84.9 ± 11.0	No difference in vitamin D status between active and inactive groups. Children with JRA who have improvement in their disease activity have an improvement in BMD heralded by an increase in serum osteocalcin values 4–87 months from disease onset.		
Harrow, UK Reeve et al. 1993 51°N [53]	JCA- treated with glucocorticoids	Prednisone n = 17 Deflazacort *n* = 17	Prednisone 10.6 ± 3.7 Deflazacort 10.3 ± 3.9	Prednisone 140.8Deflazacort 115.6	25(OH)D was surprisingly high, there was no difference between the two groups *p* = 0.8.		
Chicago, Illinois Reed et al. 1990 40°N [57]	Chronic Rheumatic Disease	*n* = 113 (82) JRA *n* = 83	1.5 to 21	Range of groups 44.9 ± 15.0 to 54.9 ± 22.5	No difference between those with active and inactive disease.		
London, UK Elsasser et al. 1982 51°N [51]	JCA	*n* = 63 serum 25(OH)D *n* = 29	Not reported	24.5 nmol/L (9 > 25 nmol/L, 20 < 25 nmol/L)	There was a marginally significant correlation between TBD and 25(OH)D concentrations (*r* = 0.37. *P* < 0.05). Only nine children had acceptable vitamin D status.		

### Vitamin D status in relation to chronic childhood arthritis classification

Twenty-one of the 38 studies (55.3%) reported 25(OH)D status for patients with JIA [21–40], eight (21.1%) for patients with JRA [41–48] and five (13.2%) for JCA patients [49–53]. Additionally, there were four studies that included patients with juvenile arthritis and other rheumatic diseases [54–57]. As the JRA classification system tended to be applied in North America and the JCA classification in Europe, there was a corresponding hemispheric-specific division in the geographic region from which JRA and JCA articles originated. Studies originated from 17 countries at latitudes ranging from 3°S to 61°N (Table 2). There were no eligible studies found that reported data below a latitude of 39°N prior to the introduction of the ILAR JIA disease classification systemic and no eligible JRA studies above 42°N.

The 2013, systematic literature review of 19 childhood arthritis studies reported vitamin D status (14 reporting 25(OH)D and 11 reporting 1,25(OH)D) suggested that at that time there was no clear link between vitamin D status and children with chronic arthritis [12]. The review also contained a meta-analysis comprising three studies that reported the prevalence of vitamin D insufficiency to be 82% in JIA [12]. Only three studies reported in the meta-analysis were conducted using ILAR JIA criteria [12].

### Comparison of study design

Seventeen studies used a cross-sectional design (*n* = 17; 44.7%) [27–30, 32–35, 37, 42, 44, 51, 53, 57–59], 16 (42.1%) a case-control design [21–26, 31, 39, 41, 43, 47–50, 60] and the remainder (5; 13.2%) were randomized controlled trials [40, 45, 46, 54, 55]. The primary objective of the majority of studies was to investigate the relationship between juvenile arthritis and bone health (*n* = 20; 52.6%). In all studies where sex distribution was reported, there were significantly more female participants than males, an observation consistent with the overall preponderance of females in JIA [61]. With the exception of one study [35], the age range of the participants was 0–21 years.

### 25(OH)D status

Of the 38 studies reviewed, six (15.8%) had mean 25(OH)D concentrations above 75 nmol/L [28, 42, 44, 45, 53, 54]. Seventeen studies (44.7%) had mean 25(OH)D concentrations between 50 and 75 nmol/L [22, 23, 25, 27, 30, 33, 34, 36, 37, 39, 40, 46–48, 55, 59] and 15 (39.5%) had values below 50 nmol/L [21, 24, 26, 29, 31, 32, 35, 38, 41, 43, 49–51, 57, 60]. Of those 15 studies, 10 reported the mean 25(OH)D value, the median value reported for one study and a range or cutoff was provided for four studies). Of the 14 studies (36.8%) that published mean 25(OH)D status of healthy control groups, nine (64.3%) had mean control values significantly greater than the population with childhood arthritis [21–23, 31, 41, 43, 49, 50], three (21.4%) had concentrations that were statistically similar [25, 39, 60], and two studies (14.3%) had mean 25(OH)D values significantly below the juvenile arthritis comparison groups [47, 48]. One study compared 25(OH)D concentrations in children with JIA to hospitalized children and found no statistically significant difference between the two groups [24]. Mean 25(OH)D status of most studies (32 of the 38; 84.2%) was below the optimal concentration of 75 nmol/L in children with arthritis [20].

### Geography in relation to 25(OH)D status in chronic childhood arthritis

**Fig. 2:**
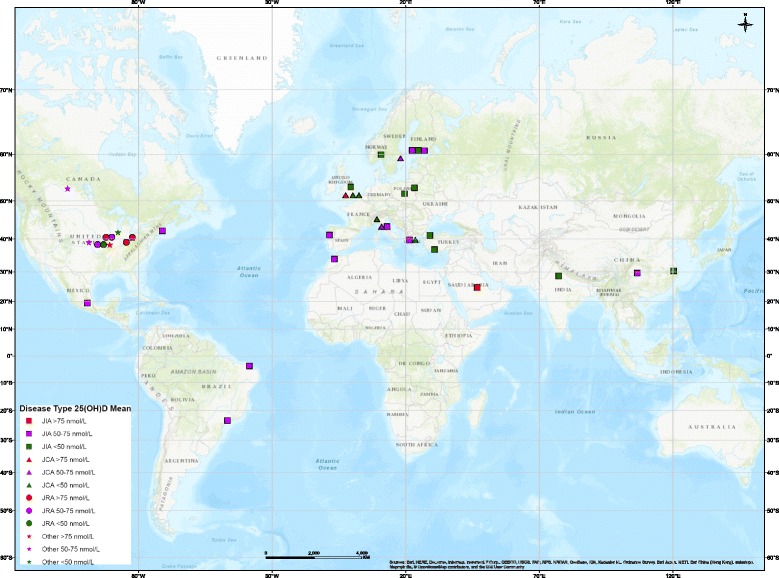
Disease type by 25(OH)D group. Note: Overlapping points are spread out. Map Produced by The Spatial Initiative, University of Saskatchewan, 2018

**Fig. 3:**
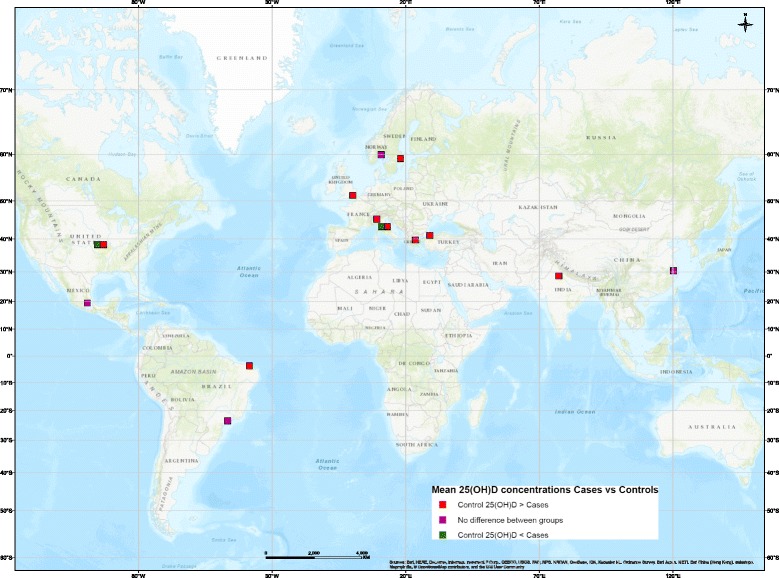
Difference in 25(OH)D concentration in active vs inactive chronic childhood arthritis patients. Note: Overlapping points are spread out. Map Produced by The Spatial Initiative, University of Saskatchewan, 2018

Vitamin D status in children with JIA appears to follow a north-south gradient (Fig. 2). While this could be due to the diagnostic resources of the countries reporting values, the gradient does appear to be present in Europe where access to care and diagnostic resources are similar. Interestingly, the relationship between reduced vitamin D status and increased disease activity also appears to be present and follow a north-south gradient (Fig. 3). More studies are required to confirm this relationship worldwide, especially in locations around the equator as well as in the southern hemisphere where thus far only two studies have taken place [22, 25].

The major source of vitamin D for most people is endogenous vitamin D synthesis induced by sunlight exposure [62]. Above 33° latitude UVB radiation is not intense enough for the cutaneous synthesis of vitamin D all year long [63, 64]. At latitudes 42° and 53^o^ North, between October to April, UVB radiation is not intense enough to elicit endogenous vitamin D synthesis [65] thus potentiating the risk of vitamin D deficiency, [63].

The prevalence of JIA, as well as the dominating subtype, varies with latitude [1]. As illustrated in Fig. 2, seven reviewed studies (18.4%) were conducted in populations residing at latitudes at or below 33° [22, 25, 27, 28, 31, 39, 60], 19 studies (50.0%) were conducted between 33 and 50° [21, 23, 30, 32, 36, 40–48, 50, 54, 55, 57, 66], and 12 at a latitude above 50° [24, 26, 29, 34, 35, 37, 38, 49, 51, 53, 59]. For those below 33°, 1 study (14.3%) reported a mean 25(OH)D concentration > 75 nmol/L, 4 (57%) reported a concentration between 50 and 75 nmol/L, and two (29%) reported values less than 50 nmol/L. For the studies that took place between 33 and 45° latitude, four studies (21.1%) reported a 25(OH)D concentration > 75 nmol/L (21%), nine (47.4%) reported a concentration between 50 and 75 nmol/L(47%) and six (31.6%) reported values less than 50 nmol/L. From the studies that took place above 45° latitude, one study (8.3%) reported a 25(OH)D concentration > 75 nmol/L, four (21.1%) reported a concentration between 50 and 75 nmol/L (33%) and seven (36.8%) reported values less than 50 nmol/L (39%).

### Current chronic childhood arthritis diagnostic criteria

Of the 21 studies that applied the current chronic childhood arthritis criteria used by ILAR to diagnose children with JIA, only one study reported mean 25(OH)D concentrations above 75 nmol/L (15%) [28]. The majority of the studies (*n* = 11; 52.4%) reported mean concentrations between 50 and 75 (52%) nmol/L [22, 23, 25, 27, 30, 33, 34, 36, 37, 40, 67], and the remaining studies (*n* = 9; 42.9%) reported a mean concentration below 50 nmol/L (43%) [21, 24, 26, 29, 31, 32, 35, 38, 60]. As latitude increased, the percentage of studies that reported mean 25(OH)D in the 50–75 nmol/L range decreased.

### Vitamin D and disease activity

No single measure has been established as an accurate indicator of childhood arthritis disease activity. While C-reactive protein and Erythrocyte Sedimentation Rate are indicators of inflammation, they alone do not fully reflect overall disease activity. In the studies reviewed, a variety of validated composite scores were used to measure function or disease activity, including the Childhood Health Assessment Questionnaire (CHAQ), the Juvenile Arthritis Disease Activity Score − 27 (JADAS-27) and American College of Rheumatology Pediatric 30 Criteria (ARC Peds 30) [68–70].

Fifteen of the 38 studies comprising this present review (39.5%) evaluated the relationship between vitamin D and disease activity (Fig. 3). Seven studies (18.4%) reported that patients with active disease or those with elevated inflammatory biomarkers had lower 25(OH)D concentrations than those patients who were in remission or who had less disease activity [23, 27, 30, 32, 35, 41, 50]. One study (26.3%) showed the opposite relationship; those with active disease had higher vitamin D concentrations than those with inactive disease [34]. Of the seven studies (18.5%) that reported no relationship between 25(OH)D and disease activity [21, 22, 25, 33, 36, 42, 57] one found a relationship between 25(OH)D concentrations and disease activity in the univariate but not the multivariate analysis [33]. Except for one study conducted in Turkey [21], all other studies conducted in Europe that explored disease activity reported an inverse association between vitamin D and disease activity.

Long-term cohort studies can further clarify the relationship between vitamin D concentration and disease duration or frequency of relapse. Such an association was explored in a cross-sectional study which found significantly reduced 25(OH)D status in JIA patients (*n* = 152) compared with 188 age-and-sex-matched controls [23]. Active disease or frequent relapse was associated with reduced vitamin D status compared to patients with no active disease or frequent flare-ups. The authors questioned whether JIA patients with more severe disease require higher supplementation of vitamin D to maintain normal 25(OH) D concentrations. As latitude increases more studies report a difference in vitamin D status between patients with active versus inactive disease in comparison to lower latitudes as illustrated in Fig. 3.

To date, the evidence to support a relationship between vitamin D and disease activity with autoimmune diseases in humans is correlative and not causative [71]. Long-term, adequately powered randomized studies which control for confounding variables (sun exposure, season, and vitamin D intake) are required to confirm a causative relationship between vitamin D and disease activity.

### Potential requirements of vitamin D intake

Vitamin D intake was only measured in seven studies [23, 26, 31, 37, 38, 43, 44]. All of these studies reported a mean or median vitamin D intake that was less than the Estimated Average Requirement (EAR) of 400 IU per day set by the IOM [19]. This is the amount of vitamin D that is expected to be sufficient for 50% of the population [19]. This indicator is used to evaluate the prevalence of inadequacy at the population level. The recommendation at the individual level is the Recommended Dietary Allowance (RDA) which ranges from 400 to 600 IU based on age groups. Two studies reported intake of vitamin D supplements by study participants but neither had a mean 25(OH)D that reached the optimal concentration [38, 44]. Three studies reported intake of both children with JIA and healthy controls [23, 26, 31]. Vitamin D intake and status was similar for two JIA patient groups [23, 26], and lower intake resulted in lower vitamin D status in the third group [31]. In comparison to the control groups, Lien et al. found similar intake and 25(OH)D status between those with JIA and controls [26]. Stagi et al. reported similar intake of vitamin D and higher 25(OH)D in controls, and in the study reported by Dey et al. the intake of control participants was two times higher than the participants with JIA and the control group had higher 25(OH)D concentrations [23, 31].

It has been theorized that there may be an increased utilization of vitamin D during active inflammation, possibly caused by \the presence of vitamin D receptor polymorphisms in patients with autoimmune diseases [6, 72]. Additional studies investigating vitamin D intake from all sources (both food and supplements) are required to determine if children with chronic arthritis require additional vitamin D to maintain serum concentrations in comparison to healthy children. Understanding this relationship will be important in the use of vitamin D as a potential adjunct therapy. Additionally, improved understanding of vitamin D needs in children with chronic arthritis will help to clarify the role of vitamin D in the underlying disease processes so that therapies that target specific vitamin D responsive immune pathways can be developed. By exploring the factors that influence vitamin D status (genetics, environment, and nutrition), we will be better able to discern an association between vitamin D and JIA.

Two articles have discussed vitamin D requirements for children with rheumatic conditions not in the context of corticosteroids. In 2011, von Scheven and Burnham suggested that in the absence of specific guidelines for children with rheumatic conditions that the American Academy of Pediatrics guidelines of 400 IU per day be used as a suggested minimum dosing regimen [4]. The authors cautioned that providing large doses of vitamin D can result in providing “too much of a good thing” and that studies comparing children with rheumatic diseases to healthy children are required. The second article was published by Vojinovic and Cimaz in 2015 and recommends that the guidelines set out by the Endocrinology Society for patients receiving corticosteroids be followed for all children with rheumatic diseases [73]. This would result in a dose of 2–3 times the current recommendation and would be approximately 2000 IU/day. This dose is still below the IOM’s tolerable upper limit for all children over the age of one (2500 IU) [19].

### Vitamin D and medication interactions in JIA patients

All but one study [24], have been conducted with children already being treated for arthritis, many receiving corticosteroids, which could impact 25(OH)D concentration and inflammatory status. Corticosteroids promote the breakdown of both 25(OH)D and 1,25(OH)D and also counteract effects of vitamin D on bone formation [74, 75]. These patients also had varying disease duration. A study of newly diagnosed individuals, however, did not compare patients with JIA to healthy controls but to hospitalized children [24] . Comparing children with JIA to healthy controls allows for discerning biologic differences that could inform treatment targets.

The lowest mean 25(OH)D concentration, 22 nmol/L (*n* = 35) was reported from India. These patients were found to be consuming significantly less vitamin D and had less sun exposure compared to healthy controls [31]. The highest 25(OH)D, 140.8 nmol/L (*n* = 17), concentrations were reported in Finland, in a population receiving Prednisone [53]. The authors hypothesized that the reason their patients’ 25(OH)D concentrations were so high was that their previous research had found low concentrations of 25(OH)D and they were encouraging their patients to consume vitamin D fortified foods and to spend time in the sun [53]. A survey of steroid-related osteoporosis, prevention and treatment practices of pediatric rheumatologists in North America was conducted by Soybilgic et al. in 2014 [76]. They found that the majority of pediatric rheumatologists are recommending vitamin D for patients who were on long-term corticosteroids [76]. The role of vitamin D in mediating bone health, especially in relation to corticosteroids, has been established [75]. Both short and long-term corticosteroid intake even at small doses impact bone health in patients with autoimmune diseases [74]. The role and an appropriate amount of vitamin D intake or 25(OH)D target for inflammation or disease activity have yet to be established.

### Additional research directions

Considering ethnicity when comparing incidence and prevalence amongst populations would be useful in understanding if the regional differences observed are due to environmental or genetic factors or a combination of the two. Evidence from a multiethnic cohort study of 1082 children at The Hospital for Sick Children, Toronto, Canada investigated the influence of ethnicity on the risk of developing JIA [77]. When the diversity of the study population was compared to that of the general Toronto region population, there was an overrepresentation of patients of European and Indigenous descent and an underrepresentation of patients of Black, Asian, or Indian subcontinent ethnicity in their cohort. European descent was significantly associated with an increased risk of developing JIA, including all subtypes except RF-positive polyarticular JIA. Exploring the environmental and genetic factors that may contribute to JIA risk in the same individual will help to clarify these findings.

While this review focused on vitamin D status in children with JIA, vitamin D may be involved in both disease development and subsequent disease activity status. Exploring elements of vitamin D status that may have a role in disease development such as early life and gestational vitamin D status as well as genes in the vitamin D pathway will help to clarify the role of vitamin D.

Season of birth has been suggested to have an impact on the risk of developing a number of autoimmune diseases such as multiple sclerosis, type 1 diabetes and celiac disease [78]. A recent study investigating month of birth and risk of JIA found a difference in the pattern of the birth month for children with JIA compared to that of the general population [78]. Children with JIA were more likely to be born between November to March, with the birth month for the general population peaking in the summer months. The study by Carlens et al. also investigated the relationship between season of birth and the risk of developing JIA and found no increased risk [79]. Season of birth may be a marker for vitamin D status in utero with children born in the non-vitamin D synthesizing periods being exposed to less vitamin D during their time in utero than those who are born during the vitamin D synthesizing seasons.

The Childhood Arthritis Risk factor Identification sTudY (CLARITY) explored the use of nutritional supplements during pregnancy and the risk of developing JIA [80]. The use of vitamin D and fish oil during pregnancy in case mothers was not significantly different from controls following covariate adjustments [80]. A case-cohort investigation from Denmark, comparing 25(OH)D status in children diagnosed with either oligoarticular or polyarticular JIA using dried blood spot samples that were collected at birth did not find any association between 25(OH)D status at birth and risk of developing JIA [81]. Concentrations of 25(OH)D fluctuated significantly by season of birth and year of birth (calendar year). There was no follow up to determine if 25(OH)D status or season during the first few months of life impacted risk of JIA or whether other subtypes of JIA were impacted by season of birth of 25(OH)D status at birth.

Certain VDR gene polymorphisms may be associated with different biologic response to vitamin D. The Cdx2 polymorphism of the VDR gene specifically the GG genotype, have been suggested to be more represented in patients with JIA compared to healthy controls who more often have the GA genotype [82]. Recently the idea of investigating epistasis (gene-gene interactions) amongst genes in the inflammatory and vitamin D pathway and how their interactions contribute to JIA risk was explored by Ellis et al. [86]. This is the first study to explore this interaction, and the authors suspect that through exploring these interactions there is the opportunity to account for the missing heritability that has been observed with complex diseases with genetic components [86]. Their work found evidence of epistasis amongst tyrosine-protein phosphatase non-receptor type 2 (PTPN2) gene and the vitamin D binding protein gene in contributing to the risk of JIA [86]. The role of genes in the vitamin D pathway on both disease development and disease activity are still in the early stages of investigation. Also how they impact the biological response involving vitamin D and inflammation remains unclear. Investigating genetic, nutritional and environmental factors that influence vitamin D in JIA could help inform ways in which vitamin D status influences the occurrence and activity of JIA. Understanding if genetic variants increase the risk of disease development will help tailor vitamin D management in individual patients and contribute to improving control of disease activity and improve outcomes.

A north-south gradient of incidence and a mechanism for the suppression of inflammation in relation to 25(OH)D status has been suggested. However, no study has summarized the current evidence of chronic childhood arthritis diagnosis by 25(OH)D status in relation to latitude and disease activity. This first step is important for the development of future studies leading to the exploration of potential optimal target concentrations of vitamin D for the reduction of inflammation in children with chronic arthritis.

### Limitations

This scoping review has limitations due to the limited amount of comparative data. Season of measurement and JIA subtype could not be considered due to a lack of reporting in most reviewed articles. With the exception of one study, all studies reviewed used various unreported types of medication in patients who had had JIA for varying durations. These variables can make it difficult to interpret the relationship between vitamin D and disease activity in relation to both inflammation status and risk of relapse. Of the studies that investigated the relationship between vitamin D and function or disease activity, various measures were used. The most common included the CHAQ, JADAS-27 and ARC Peds 30. The JADAS-27 and ACRS Peds 30 both include active joint counts in their scoring which confounds comparisons of disease activity between patients with different JIA that are defined by numbers of joints involved. Subtypes [17, 83, 84]. Many studies included multiple subtypes of JIA measured by the same disease activity score that included an active joint count. This review was unable to explore the relationship between vitamin D status and ethnicity, vitamin D receptor genes or other genes that influence vitamin D metabolism.

## Conclusion

This is the first scoping review to summarize research relating to vitamin D and JIA in the context of vitamin D status, latitude, disease activity. It is also the first to map the results according to geography. Thirty-two studies (84.2%) reported a mean 25(OH)D concentration below 75 nmol/L or the optimal value. This suggests that whether due to inadequate intake or increased utilization the majority of children with juvenile arthritis do not have optimal 25(OH)D status as defined by the Endocrine Society [20]. The optimal concentration of 25(OH)D and the corresponding dietary requirements for patients with chronic childhood arthritis has yet to be determined. Further, the relationship between vitamin D status and disease activity in children with JIA is still unclear. Studying newly diagnosed patients who are treatment naive for longer periods of time would help characterize this relationship as there would be fewer confounders associated with patients who have had the disease for varying durations (medication, lifestyle modifications, and disease duration). Thus far, we know that there is a role for vitamin D in the inflammatory pathways, a high prevalence of 25(OH)D insufficiency among children with JIA, and an established link of vitamin D with other autoimmune diseases. We do not, however, know the optimal vitamin D status for children with JIA, whether reduced vitamin D is caused by increased utilization or reduced vitamin D status in children with JIA, the impact of vitamin D in disease activity or the role of VDR polymorphisms with JIA. Larger, long-term studies of new-onset JIA are required to explore the association. The relationship between vitamin D status and JIA over time in newly diagnosed individuals has yet to be investigated. Investigating the genetic and environmental role that vitamin D plays in the prevention and control of JIA in the same children will help to tease out the multifaceted role played by vitamin D in this disease. Being able to suggest specific targets for vitamin D status as a potential adjunct therapy in the treatment of JIA and understanding how genetic variants increase the risk of disease development will enhance the quality of life of patients and their families.

## Abbreviations

1,25(OH)2D): 1,25-dihydroxyvitamin D
25(OH)D: 25-dihydroxyvitamin D
CINHAL: Cumulative Index to Nursing and Allied Health Literature
CLARITY: Childhood Arthritis Risk factor Identification sTudY
CYP27B1: Cytochrome p450 27B1
GIS: Geographic Information Systems
IOM: Institute of Medicine
JCA: Juvenile Chronic Arthritis
JIA: Juvenile Idiopathic Arthritis
JRA: Juvenile Rheumatoid Arthritis
MeSH: Medical Subject Heading
VDR: Vitamin D receptor
